# The intersection molecule MDA5 in Cancer and COVID-19

**DOI:** 10.3389/fimmu.2022.963051

**Published:** 2022-08-31

**Authors:** Renjing Jin, Xiaoqing Cao, Mingjun Lu, Qing Gao, Teng Ma

**Affiliations:** ^1^ Cancer Research Center, Beijing Chest Hospital, Capital Medical University, Beijing Tuberculosis and Thoracic Tumor Research Institute, Beijing, China; ^2^ Department of Thoracic Surgery, Beijing Chest Hospital, Capital Medical University, Beijing Tuberculosis and Thoracic Tumor Research Institute, Beijing, China

**Keywords:** MDA5, pattern recognition receptors, SARS-CoV-2, anti-tumor immunity, cGAS-STING

## Abstract

The connections between pattern recognition receptors (PRRs) and pathogen-associated molecular patterns (PAMPs) constitutes the crucial signaling pathways in the innate immune system. Cytoplasmic nucleic acid sensor melanoma differentiation-associated gene 5 (MDA5) serves as an important pattern recognition receptor in the innate immune system by recognizing viral RNA. MDA5 also plays a role in identifying the cytoplasmic RNA from damaged, dead cancer cells or autoimmune diseases. MDA5’s recognition of RNA triggers innate immune responses, induces interferon (IFN) response and a series of subsequent signaling pathways to produce immunomodulatory factors and inflammatory cytokines. Here we review the latest progress of MDA5 functions in triggering anti-tumor immunity by sensing cytoplasmic dsRNA, and recognizing SARS-CoV-2 virus infection for antiviral response, in which the virus utilizes multiple ways to evade the host defense mechanism.

## Introduction

The innate immune system provides the first-line defense against pathogen infection. Among them, PRRs can detect PAMPs and damage associated molecular patterns (DAMPs). In mammals, a series of nucleic acid sensors (NSs) can be used as PRRs to recognize the nucleic acid fragments released when the virus attacks the human body, transduce signals to induce host defense response. The innate immune system includes several important NSs, including cyclic guanosine-monophosphate adenosine-monophosphate synthase (cGAS), absent in melanoma 2 (AIM2) and Toll-like receptor 9 (TLR9) that sense DNA fragments, and RIG-I like receptors (RLRs), nucleotide-binding oligomerization domains (NODs), Toll-like receptor 3 and 7 (TLR3 and TLR7) that sense RNA fragments (1, 2). In the process of RNA-dependent RNA replication and synthesis, the RNA viruses will produce double-stranded RNA (dsRNA). Some DNA viruses also produce dsRNA in their lifetime (3). In earlier years, studies have found a cytosolic sensor, dsRNA-dependent protein kinase (PKR), can bind dsRNA in its N-terminal regulatory region to block dsRNA formation, preventing the synthesis of viral proteins (4). Similarly, viral dsRNA can also activate 2’-5’-oligoadenylate Synthetase (OAS) directly, which activates the anti-viral endonuclease RNase L, thus preventing RNA virus replication (5, 6). Therefore, dsRNA can function as PAMP in the process of virus infection.

There are two classical ways to recognize viral dsRNA in the innate immune system. TLR3 mainly senses the dsRNA locates in the endosome, including dsRNA produced by lysis or necrosis from the virus infects cells, and dsRNA internalized by receptor-mediated endocytosis (7–10). TLR3 signaling activates IRF3/IRF7, which mediates IFN-α/β and a series of IFN-induced genes (11–13). RLRs bind specifically to RNA fragments produced by pathogenic microorganisms or self-produced RNA fragments in cytoplasm, and initiate immediate immune effects (7). RLRs mainly include retinoic acid-induced genes (RIG-1, also known as DDX58), MDA5 (also known as IFIH1) and laboratory of genetics and physiology 2 (LGP2). These proteins are usually expressed at a low level. RIG-I and MDA5 interact with interferon-beta promoter stimulator 1 (IPS-1, also known as MAVS, VISA, Cardif) in the outer membrane of mitochondria, which transmits signals to IFN-β and downstream interferon-stimulated genes (ISGs) through transcription factor interferon regulatory factor 3 (IRF3) and IRF7 and nuclear factor (NF)-κB (14–17). Currently, the mechanism of innate immune response induced by RIG-1 has been thoroughly studied, but the mechanisms of MDA5 in cancer and virus infection are to be understood.

Latest data show that in COVID-19, SARS-CoV-2 replicates in lung epithelial cells and induces delayed interferon (IFN) response, in which MDA5 acts as the main sensor to recognize SARS-CoV-2 infection and trigger antiviral response (18). Unfortunately, viruses can either disturb key regulatory structures, or interfere with interferon (IFN) system in multiple ways, so as to escape the body’s defense mechanism. This review briefly summarizes the intersection role of MDA5 in antitumor immune response and SARS-CoV-2 innate immune signaling pathway, hoping to provide a deeper understanding of the molecular basis of these diseases.

## Nucleic acid sensing pathway

### RIG-I-like receptors family triggers interferon-related signal response

RLRs consists of three members: RIG-I, MDA5 and LGP2 (19). All members of the family have a core tandem DExD/H-box helicase domain and a C-terminal domain (CTD), which jointly trigger and integrate RNA signaling pathways (1). In addition, RIG-I and MDA5 also have two N-terminal caspase activation and recruitment domains (CARD), which mediate downstream signal transduction. After binding to RNA, RIG-I and MDA5 promote the transcription of IFN and other antiviral or immunomodulatory genes (20). On this basis, type I IFN can further induce RIG-I and MDA5 at the transcriptional level through a positive feedback loop, and amplify the interferon response (21). Additionally, several negative regulatory factors are involved to prevent interferon self-enhancement, including 2’-phosphodiesterase which degrades 2-5A, IFN-β-induced inhibitor LGP2 and ubiquitin ligase RNF125 (22, 23). LGP2, the third member of the RLR family, mainly plays roles in regulating RIG-I and MDA5. Though LGP2 does not function through MAVS, the interaction between MDA5 and LGP2 enhances the perception of some RNA viruses, especially by limiting RIG-I signals to enhance MDA5 signals (24). Structurally, LGP2 promotes the rapid formation of short microfilaments of MDA5-dsRNA, which eventually leads to the enhancement of downstream signals and the increase of type I IFN responses (25). IFN shows antiviral activities at multiple levels, mainly including viral entry, viral polymerase function, host cell translation, RNA stability, particle budding, apoptosis, and generally enhanced innate and adaptive immune responses (26).

Furthermore, RLRs not only induce IFN responses, but also trigger apoptosis, pyroptosis and necroptosis (27–29). These pathways can clear damaged cells in autoimmune diseases and attacked by cytotoxic signals, further contributing to the release of cytokines and inflammatory mediators.

### Melanoma differentiation-associated gene 5

MDA5 gene was first reported to be induced by differentiation, cancer reversal and apoptosis in 2002 (30). MDA5 protein is located in the cytoplasm, induced and regulated by IFN-β. It has been proved to be the double-stranded RNA-dependent ATPase. Later, it was proposed for the first time that MDA5 harbors a CARD domain and an RNA helicase motif (30, 31). Follow-up studies demonstrate that MDA5 can bind to viral dsRNA as well as its synthetic analogue Poly (I:C), and then mediate type I interferon response. The overexpression of MDA5 could enhance the type I interferon response stimulated by transfection of Poly (I:C) (32). The above findings demonstrate that MDA5 is an RNA sensor that can induce type I interferon.

Though MDA5 and RIG-I share similar domains and signal pathways, MDA5 has selective specificity in recognizing dsRNA. It has been reported that MDA5 mainly recognizes the double-stranded structure within dsRNA by using different orientations of CTD structure. MDA5 distributes along dsRNA by direct protein-protein contact, and the tandem CARD structure decorated outside the MDA5 core filaments must be oligomerized to activate the slender structure of the adapter protein MAVS, which is found to be particularly stable on long dsRNA molecules. Some researchers have also found that MDA5 requires an “RNA network”, not a simple long-stranded dsRNA molecule, but a high-order RNA structure containing single-stranded RNA and long-stranded dsRNA (33). Therefore, it is also important to determine the specific RNA fragment structure MDA5 chooses to play an accurate role (34). Previous study showed that RIG-I responds to viral RNA containing triphosphate, which connects the 5 ‘end to the base pairing region of the blunt-end (35). Later it was found that RIG-I mediates an antiviral response to RNA containing 5-diphosphate (36). Either *in vitro* transcribed RNA or chemically synthesized, 5-diphosphate RNA can act as an RIG-I agonist, which is an important means of recognition in the innate immune system. But this recognition mode usually exists in some viral RNA (36). At present, the specific RNA that can bind to MDA5 is not clear. It is necessary to find out the characteristics of MDA5 stimulating RNAs, thereby explaining why MDA5 function is different among antiviral immunity, autoimmunity, autoinflammatory disease and anti-tumor immune process.

MDA5 not only functions as nucleic acid sensor of exogenous dsRNA, but also plays an important role in detecting abnormal dsRNA fragments from endogenous sources. In recent years, cancer therapy targeting epigenetic suppressors has become a hot spot, which activates reverse transcription factors in the human genome (37). At the same time, the use of viral mimicry in cancer therapy can cause cancer cells to lose their adaptability and stimulate innate and adaptive immune responses. When endogenous immunogenic reverse transcriptional elements such as Alu elements are activated and mitochondrial enzymes SUV3 and PNPase involved in mitochondrial RNA degradation are depleted or mutated, mitochondrial dsRNA escapes to the cytoplasm, triggering MDA5-dependent anti-abnormal nucleic acid signaling pathways (38). These processes prove that endogenous dsRNA fragments can also trigger MDA5 and mediate cytokine- IFN immune pathway.

In addition to its antiviral protective effects, MDA5 is associated with autoimmune diseases such as type 1 diabetes (T1D) or systemic lupus erythematosus (SLE), which may be associated with chronic induction of type I interferon, leading to initiation or enhancement of autoinflammation and autoimmune condition (39). Mutation of IFIH1 gene encoding MDA5 can lead to a rare neurological disorder Aicardi–Goutières syndrome (AGS), which is characterized by abnormal production of type I IFN. The mutation of MDA5 gene can lead to the substitution of single amino acids in the helicase domain of MDA5 (40, 41). MDA5-mediated auto-inflammatory diseases may indicate the importance of MDA5 in perceiving the specific RNA elements.

## Anti-tumor strategies based on MDA5 immune pathway

**Figure 1:**
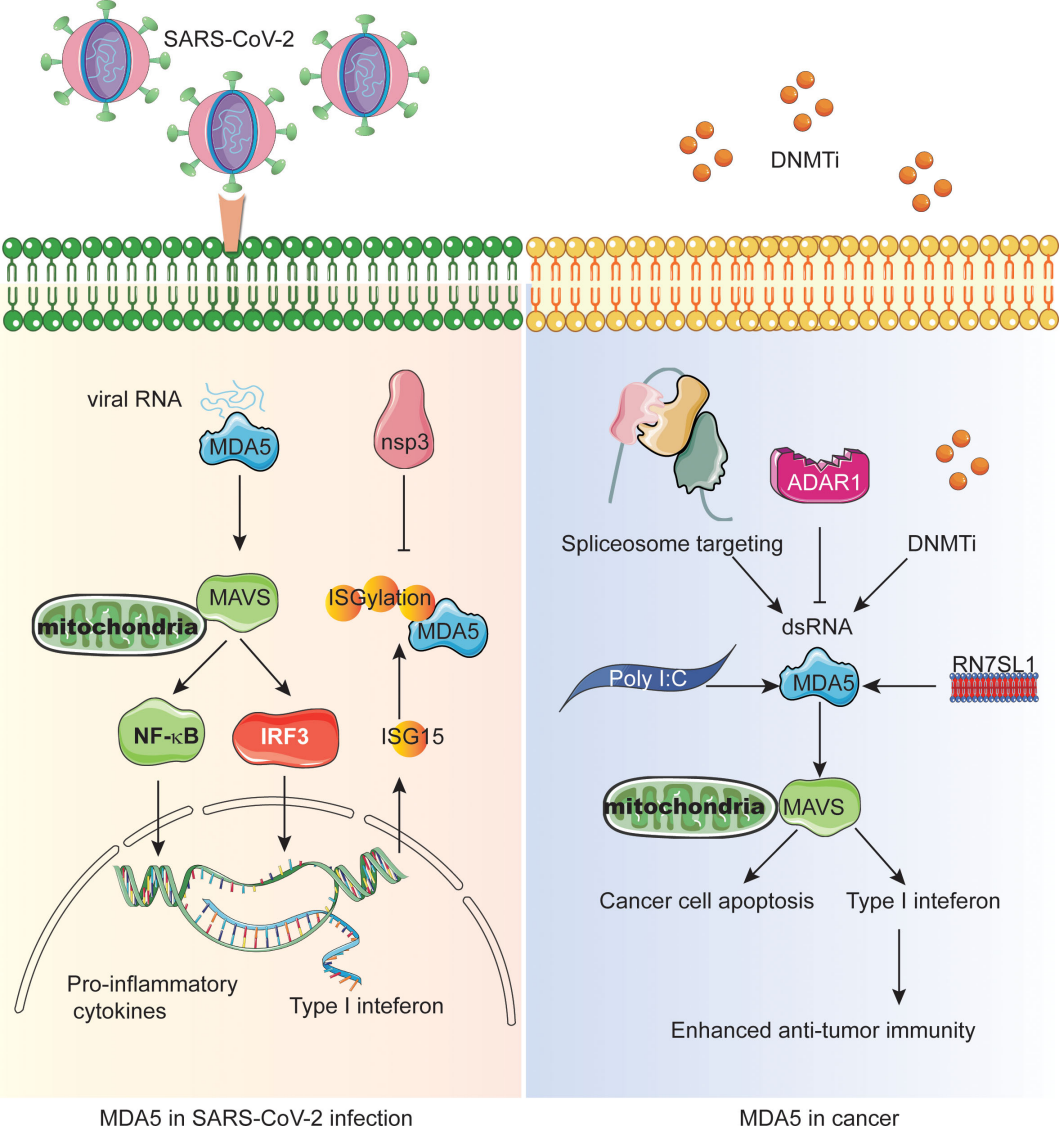
The central role of MDA5 in SARS-CoV-2 infection and interferon-mediated anti-tumor immunity. The left panel shows the MDA5-MAVS-NFκB/IRF3 signaling during SARS-CoV-2 infection and how the nonstructural proteins inactivate MDA5 through deISGylation. The right panel shows the different mechanisms resulting in endogenous dsRNA production or MDA5 activation in cancer cells which enhances the antitumor immunity and/or cancer cell apoptosis.

Tumor cells share common key characteristics, such as oxidative stress, genomic instability and mutation, and changes in metabolic rate, which can lead to nuclear or mitochondrial DNA damage, thus releasing damaged nucleic acid fragments into the cytoplasm (42). In mammalian cells, two typical cytoplasmic nucleic acid sensing pathways are cGAS-stimulator of interferon genes (STING) and RLRs-MAVS pathways, which are responsible for the recognition of cytoplasmic DNA and RNA, respectively (43, 44). MDA5 as cytoplasmic dsRNA sensor recognizes long-chain high-order dsRNA structures (45). The MDA5 signaling pathway mainly interacts with the adapter MAVS and recruits TBK1. The phosphorylation cascade allows signal transduction to lead to the production of IRF3 and IRF7, which usually leads to the production of IFNs, which subsequently induces the activation of ISGs and NF-κB target genes (**Figure 1**) (20).

The most widely used tumor treatment strategy is the use of anti-tumor DNA demethylating agents and DNA methyltransferase inhibitors (DNMTis) (46, 47). Demethylation in the normal suppressed region of the genome can lead to endogenous retrovirus transcription, triggering cytoplasmic dsRNA sensing in cancer cells to activate MDA5 and MAVS, resulting in reduced cell growth and self-renewal, thus mimicking viral infection (**Figure 1**) (48, 49). In addition, DNMTis treatment enhanced anti-CTLA-4 immune checkpoint therapy in preclinical melanoma models. Similarly, telomerase reverse transcriptase (TERT) can activate endogenous retroviruses (ERV) independent of its telomerase activity to form double-stranded RNA, which is sensed by the MDA5-MAVS pathway and triggers IFN signals in tumor cells (50, 51). However, ERV and IFN signal stimulate the infiltration of suppressor T cells at the same time, indicating that IFN signal mediated by TERT accounts for tumor immunosuppression. Significantly, TERT is considered to be a marker of tumor cells because of the wide area of activation during tumorigenesis.

Surprisingly, the activation of NSs can change the tumor microenvironment (TME) from immunosuppressive state to pro-inflammatory state (52–54). However, some studies have shown that PRRs and IFN signals promote tumor progression or immune tolerance, but not pro-inflammatory effect in immune cells (55), indicating that we need to target the PRRs of immune cells in TME in the future to improve the efficacy. Therefore, the CAR-T cells were engineered to produce a non-coding RNA called ‘*RN7SL1*’ as a new PRR agonist (**Figure 1**). Normally, *RN7SL1* can be shielded from being recognized by PRR, but when it is not shielded, it can mimic viral RNA and activate nucleic acid sensors MDA5 and RIG-I, thus enhancing endogenous anti-tumor immunity (54, 56).

Since MDA5 dominates the perception of synthetic dsRNA analogue poly (I:C) and triggers the cytoplasmic interferon response (**Figure 1**). Studies on triple negative breast cancer (TNBC) have shown that Poly I:C inhibits transforming growth factor-β (TGF-β) signal transduction in a MDA5 or RIG-I-dependent manner, thus promoting cancer cell death, and this effect can be weakened by forced expression of Smad3 (57). Since TGF- β is the characteristic of promoting the migration, invasion, bone metastasis and survival of tumor cells in TNBC, inhibition of TGF-β may be an effective strategy for the treatment of metastatic cancer. Interestingly, spliceosome-targeted therapies can also trigger an antiviral immune response in TNBC through dsRNA formation from mis-spliced RNA (**Figure 1**) (58).

Epigenetic inhibitors for cancer therapy can activate reverse transcription factors in the human genome, but the clinical efficacy of epigenetic therapy is currently limited. Viral mimicry can cause cancer cells to lose their adaptability and stimulate innate and adaptive immune responses (59–61). In order to find targets for synergistic action with viral mimic response, many researchers have focused on immunogenic reverse transcriptional elements activated by epigenetic therapy. Intronic and intergenic SINE elements, especially inverted-repeat Alus, are the main sources of drug-induced immunogenic dsRNA (37). In mammals, ADAR1, an RNA editing enzyme characterized by binding to endogenous dsRNA and converting adenosine to inosine (A to I), targets and destroys inverted-repeat Alu dsRNA, thereby preventing MDA5 activation (**Figure 1**). In addition, some studies have shown that the deletion of ADAR1 is highly sensitive to tumor immunotherapy, overcoming the resistance of immune checkpoint blockade (ICB), and the deletion of ADAR1 can reduce the A to I editing of endogenous dsRNA, triggering MDA5 and PKR interferon-dependent anti-tumor response, subsequent tumor growth inhibition and pro-inflammation (62). The deletion of ADAR1 overcomes the resistance to PD-1 checkpoint blockade caused by antigen presentation inactivation of tumor cells (62–64). Consuming ADAR1 in cancer cells can break the negative feedback loop, inhibit tumor growth and reduce the incidence of cancer. A study showed that an *FBXW7* inactivating mutation from a patient with melanoma is associated with resistance to PD-1 blockade (65). Both MDA5 and RIG-I are required for *Fbxw7*-dependent regulation of Type I interferon signaling and immune microenvironment. Therefore, combining epigenetic therapy with anti-tumor immunity will be promising treatment option of cancer.

In melanoma cells, RIG-I and MDA5 also initiate a p53-independent Noxa pro-apoptotic signal pathway, which is independent of type I IFN response (61, 66, 67). Although this pro-apoptotic signal pathway is also active in non-malignant cells, the sensitivity of these cells to apoptosis is much lower than that of melanoma cells. Endogenous Bcl-xL can block RIG-I and MDA5-mediated apoptosis in non-malignant cells. Both RIG-I and MDA5 ligands can also reduce the lung metastasis of human tumor in immunodeficient mice. These results confirm that RIG-I and MDA5 ligands have therapeutic potential in solid tumors through inducing tumor cells to apoptosis.

In general, targeting MDA5 signaling pathway has promising advantages in cancer immunotherapy.

## MDA5 in SARS-CoV-2 virus infection

### SARS-CoV-2

SARS-CoV-2 (severe acute respiratory syndrome coronavirus 2) has been identified as the pathogen of the major epidemic COVID-19 (67). SARS-CoV-2 is an enveloped, positive single-stranded RNA β coronavirus. The diameter ranges from 80 to 160 nm. The virion contains a positive-stranded single-stranded RNA genome of about 30kb, with a 5’-cap structure and a 3’-poly (A) tail (68, 69). It usually consists of 15 open reading frames (ORF) that encode 29 proteins. Its genome can be divided into two parts, the region encoding non-structural proteins (ORF1a and ORF1ab) and the region encoding conserved structural proteins. The former accounts for about 2/3 of the total length of the genome, while the latter accounts for the rest. ORF1a/ORF1ab was translated into poly-protein 1a (pp1a) and poly-protein 1ab (pp1ab) respectively. pp1a is cleaved into 11 nonstructural proteins (nsps) by papain (PLpro) and main protease (3CLPRO), while pp1ab is cleaved into 16nsps. 3’-terminal conserved structural protein region contains 13 ORFs and encodes four main structural proteins, namely spike (S) protein, envelope (E) protein, membrane (M) protein and nucleocapsid (N) protein.

SARS-CoV-2 replicates mainly in the ciliated cells of the nose and bronchioles and type 2 lung cells in the alveolar region, as well as in the gastrointestinal tract. After SARS-CoV-2 binds to the host receptor angiotensin converting enzyme-2 (ACE2) to facilitate entry, viral RNA is translated into replication complexes, resulting in genome replication and the production of offspring virions (70). COVID-19’s extrapulmonary symptoms are thought to be mediated by ACE2 receptor on small vascular endothelium, intestinal tract, smooth muscle and skeletal muscle (71). It has been reported that SARS-CoV-2 is similar to other coronaviruses in that dsRNA intermediates are produced during virus replication, and these intermediates can be recognized by RNA PRRs- MAVS cascade, or the TLRs family, especially TLR3 which activates the TRIF to induce IFN-β (70).

### MDA5-dependent anti-SARS-CoV-2 response

Through the in-depth study of the response of host cells to SARS-CoV-2 infection in primary human airway epithelial cell line (HAE) and immortalized lung cell line, it was proved for the first time that MDA5 is the main sensor for recognizing SARS-CoV-2 infection in lung cells (72). In the study of lung cancer cell line Calu-3, it was also found that human pulmonary epithelial cells mainly mediated strong antiviral response through MDA5 sensor, which led to delayed induction of type I and III interferon signals, and finally led to the restriction of virus replication (70).

These two types of IFN bind to their respective receptors (IFNAR) in an autocrine or paracrine manner and trigger the activation of the Janus kinase (JAK), which activates and phosphorylates signal transducers and transcription factors STAT1 and STAT2 pathway, leading to the expression of ISGs (26, 73). These genes exert antiviral activity directly or indirectly through different mechanisms. Although the specific molecular mechanism of SARS-CoV-2-mediated IFN remains to be studied, IFN signaling is important in immunopathology.

According to transcriptome analysis, the increase of cytokines and interferon signals in the lungs of patients with COVID-19 is usually induced by transcription factor NF-κB (74). NF-κB can be activated downstream of MAVS or activated by other PRRs. In the process of recognizing SARS-CoV-2 in lung epithelial cells, the biological functions of LGP2 and NOD1 have been proved to be essential, but RIG-I has not been found to recognize virus and induce interferon response (18). One study also showed that RNA extracted from SARS-CoV-2-infected VeroE6 cells activated MDA5 but not RIG-I after transfection of human lung fibroblasts (75). At the same time, it is reported that both MDA5 and RIG-I can sense SARS-CoV-2 infection in Calu-3 cells (76). Surprisingly, in A549, HCT116 and THP1 cells, SARS-CoV-2 virus infection and replication neither take place, nor up-regulate the response of antiviral cytokines (77).

### SARS-CoV-2 evades host antiviral response

It is worth noting that the absence of sensors did not significantly increase the level of viral RNA or the number of infectious viruses released, indicating that innate immune activation through RNA sensing did not effectively inhibit viral replication (76). Indeed, it has been reported that SARS-CoV-2 triggers a MDA5-dependent interferon response, but this response does not control viral replication in primary HAE cells and pulmonary epithelial cells (72). The follow-up study showed that type I IFN pretreatment could effectively inhibit SARS-CoV-2 replication.

SARS-CoV-2 evades the host defense not only through replication dynamics, but also the downstream immune factors of IFN signaling pathways. The activation of RLRs induces the production of IFN and then transmits antiviral signals by up-regulating ISGs. ISG15, a ubiquitin-like protein can covalently bind to the lysine residues of the target protein, a process known as ISGylation or ISG modification. At the same time, the binding of ISG15 is indispensable for the antiviral IFN response mediated by viral RNA sensor MDA5 (75). Modification of CARD structure of MDA5 by ISG promotes its oligomerization, which triggers a series of innate immune activation against SARS-CoV-2. However, direct de-ISGylation mediated by the papain-like protease (PLpro) domain of nsp3 in SARS-CoV- 2 can antagonize ISG15-dependent MDA5 activation and has been shown to be a key escape mechanism (**Figure 1**, **Table 1**).

The membrane protein (M) in SARS-CoV-2 structure has been shown to inhibit the expression of IFN-β and interferon-stimulated genes induced by RIG-I, MDA5, IKK-ϵ and TBK1, as well as IRF3 phosphorylation and dimerization induced by TBK1 (**Table 1**) (79). M protein not only interacts with MDA5 and TBK1, but also inhibits the production of type I interferon by ubiquitin degradation of TBK1 connected by K48, but its potential molecular mechanism is not completely clear.

Viral RNA-dependent RNA polymerase (RdRp) nsp12 can attenuate the activation of IFN-β promoter induced by SARS-CoV-2 virus or Poly (I:C) in a dose-dependent manner (78). It can also inhibit the activation of interferon promoter triggered by overexpression of RIG-I, MDA5, MAVS and IRF3 (**Table 1**). Although it does not affect the phosphorylation of IRF3, it inhibits IRF3 nuclear translocation, which is independent of the activity of nsp12 polymerase.

Open reading frames 9b (ORF9b) in SARS-CoV-2 structure inhibits the production of type I and type III interferons by targeting multiple molecules of cytosolic dsRNA antiviral signaling pathways, including RIG-I/MDA5-MAVS, TLR3-TRIF and cGAS-STING signaling pathways, and finally negatively regulates antiviral immunity and promotes viral replication (**Table 1**) (80).

**Table 1:** SARS-CoV-2 proteins involved in MDA5 mediated antiviral immunity.

Protein Description	Function	Reference
**N**on-structural proteins		
nsp3	de-ISGylation of MDA5 mediated by the papain-like protease (PLpro) domain	(75)
nsp12	attenuates the activation of IFN-β promoter	(78)
**S**tructural proteins		
M protein	Interacts with MDA5 and TBK1; Induces ubiquitin degradation of TBK1	(79)
Accessory proteins		
Orf9b	Targeting RIG-I/MDA5-MAVS, TLR3-TRIF and cGAS-STING signaling pathways	(80)

There is data that redefined the characteristics of COVID-19 through in-depth analysis of SARS-CoV-2 transcriptional response, and it showed that SARS-CoV-2 replication caused low levels of type I or III IFN response and high levels of chemokines and inflammation (81, 82). However, expression of exogenous virus receptor ACE2 in A549 cells and CALU-3 cells can lead to strong virus replication and obvious production of type I or III IFN. Inflammatory mediators produced during epithelial cell infection can stimulate primary macrophages to enhance the production of cytokines and driver cell activation. Therefore, for the treatment of COVID-19, it is difficult to negate the start with inflammatory factors. It is more widely understood that early IFN responses are protective and beneficial whereas late IFN responses are pathological/inflammatory (82).

## Conclusion

In summary, mammalian cytosolic nucleic acid sensing plays a central role in inducing innate and adaptive immune responses to tumors and viruses, and triggering the innate immune system helps to combat the induced immunosuppressive response. This review describes that MDA5 can recognize cytoplasmic dsRNA for cytoplasmic immune response, and the application of MDA5 agonists may be a new strategy for cancer immunotherapy. However, the role of MDA5-MAVS pathway in tumor immunity is still very complex, and many questions remain unanswered. Recently, increasing evidences have illustrated how the innate immune pathway in the tumor microenvironment changes tumorigenesis in different tumors, thus affecting the subsequent design of effective immunotherapy trials (83, 84).

In addition, MDA5 is the main sensor to SARS-CoV-2 infection, but whether RIG-I and other pathogen recognition sensors can help the host against virus and the further sensing mechanism of SARS-CoV-2 in lung epithelial cells remains to be studied. So far, the literature reports have shown us the immunopathology of SARS-CoV-2 and the mechanism of immune escape. Future efforts will focus on elucidation of the virus invading the host and the comprehensive stimulation of the immune system, and the formation of inflammatory microenvironment. Understanding of both the pre-existing inflammatory environment of the host and the extensive impact caused by many evasive factors of the virus itself will facilitate implementation of targeted therapies and development of effective treatments from many aspects.

## Author contributions

Concept and design: TM. Administrative support: QG and ML. Collection and assembly of data: RJ and XC. Data analysis and interpretation: all authors. Manuscript writing: all authors. Final approval of manuscript: all authors.

## Funding

This study was support by Beijing Xisike Clinical Oncology Research Foundation ( Grant No. Y-HR2020MS-0156).

## Conflict of interest

The authors declare that the research was conducted in the absence of any commercial or financial relationships that could be construed as a potential conflict of interest.

## Publisher’s note

All claims expressed in this article are solely those of the authors and do not necessarily represent those of their affiliated organizations, or those of the publisher, the editors and the reviewers. Any product that may be evaluated in this article, or claim that may be made by its manufacturer, is not guaranteed or endorsed by the publisher.
